# Clonal Expansion of New Penicillin-Resistant Clade of *Neisseria meningitidis* Serogroup W Clonal Complex 11, Australia

**DOI:** 10.3201/eid2308.170259

**Published:** 2017-08

**Authors:** Shakeel Mowlaboccus, Keith A. Jolley, James E. Bray, Stanley Pang, Yung Thin Lee, Jane D. Bew, David J. Speers, Anthony D. Keil, Geoffrey W. Coombs, Charlene M. Kahler

**Affiliations:** University of Western Australia, Perth, Western Australia, Australia (S. Mowlaboccus, D.J. Speers, C.M. Kahler);; University of Oxford, Oxford, UK (K.A. Jolley, J.E. Bray);; Murdoch University, Murdoch, Western Australia, Australia (S. Pang, Y.T. Lee, G.W. Coombs);; Fiona Stanley Hospital, Perth (S. Pang, G.W. Coombs);; Queen Elizabeth II Medical Centre, Perth (J.D. Bew, D.J. Speers);; Princess Margaret Hospital for Children, Perth (A.D. Keil);; Telethon Kids Institute, Perth (C.M. Kahler)

**Keywords:** Neisseria meningitidis, bacteria, serogroup W, penicillin resistance, antimicrobial resistance, clonal complex 11, clonal expansion, meningococcal disease, meningitis/encephalitis, core-genome analysis, Western Australia, Australia

## Abstract

In Western Australia, *Neisseria meningitidis* serogroup W clonal complex 11 became the predominant cause of invasive meningococcal disease in 2016. We used core-genome analysis to show emergence of a penicillin-resistant clade that had the *penA_253* allele. This new penicillin-resistant clade might affect treatment regimens for this disease.

Invasive meningococcal disease (IMD) is caused by a meningococcus, *Neisseria meningitidis*. The main manifestations of this disease are septicemia or meningitis. Meningococcal strains can be classified into 12 serogroups phenotypically and into sequence types (STs) by multilocus sequence typing (*1*). Similar STs are grouped into the same clonal complex (cc). IMD is most commonly caused by isolates expressing a serogroup A, B, C, W, X, or Y polysaccharide capsule. Until recently, serogroup A was the major cause of disease in Africa (*2*). Serogroups B, C, and Y continue to predominate in the United States, Europe, Asia, and Australia (*3*,*4*).

In Australia, after introduction of serogroup C conjugate vaccine in the national immunization program in 2003, incidence of serogroup C has decreased; serogroup B predominated during 2004–2015. However, during 2016, the prevalence of serogroup W disease increased because of *N. meningitidis* strains in the cc11 lineage (MenW:cc11) (*5**,**6*), which have also been reported worldwide. Although extensive core-genome analyses of these MenW:cc11 strains have been reported (*7*,*8*), antimicrobial drug susceptibility of these clinical isolates has not been generally reported.

Although penicillin has been used for control of IMD, clinical isolates relatively resistant to this drug have been reported worldwide. For meningococci, a penicillin MIC >2 mg/L is caused by plasmid-mediated β-lactamase-production but is extremely rare (*9*). Conversely, isolates conferring intermediate resistance to penicillin (MIC 0.12–0.25 mg/L) are uncommon but the frequency of these isolates varies geographically. The mechanism of relative resistance in these isolates involves expression of altered forms of 1 of 4 penicillin-binding proteins (PBPs) that are involved in peptidoglycan biosynthesis during bacterial growth and cell division (*10*).

Although treatment with penicillin is still effective against these penicillin-intermediate strains, low-dose treatment regimens may fail for cases involving penicillin-resistant isolates (MIC >0.5 mg/L) (*11*). We report recent emergence and clonal expansion of a phylogenetically related cluster of penicillin-resistant MenW:cc11 isolates in Western Australia.

## The Study

Western Australia is the largest state in Australia (land area 1.02 million square miles). However, it has a population of only 2.5 million persons. In concordance with the national trend, there has been a shift in the predominant serogroup in Western Australia; MenW was responsible for most IMD cases in 2016. The first laboratory-confirmed MenW:cc11 case in Western Australia was recorded in April 2013 and was the only MenW case for that year. Since that time, an additional 18 MenW:cc11 laboratory-confirmed cases have been reported, representing 11% (n = 2) of all IMD cases in 2014, 27% (n = 3) in 2015, and 67% (n = 13) in 2016, a significant increase from 2014 through 2016 (p = 0.0004, by Fisher exact test). Three deaths were caused by MenW:cc11 infection, 1 in 2015 and 2 in 2016.

The 19 MenW:cc11 strains isolated during January 1, 2013–December 31, 2016, were assessed for susceptibility to penicillin, ciprofloxacin, ceftriaxone, and rifampin. We performed drug susceptibility testing by using the Etest (bioMérieux, Marcy l’Etoile, France). MIC results were interpreted according to Clinical Laboratory Standard Institute (http://clsi.org) breakpoints. All isolates were susceptible to ciprofloxacin (MIC <0.03 mg/L), ceftriaxone (<0.12 mg/L), and rifampin (<0.5 mg/L). However, variation in penicillin susceptibility was observed: 8 were susceptible (<0.06 mg/L), 2 were less susceptible (0.12–0.25 mg/L), and 9 were resistant (>0.5 mg/L). All isolates less susceptible to or resistant to penicillin were identified in 2016.

We further characterized isolates by using whole-genome sequencing with the Miseq Platform (Illumina, San Diego, CA, USA). Raw reads were assembled, auto-tagged, and curated by using the BIGSdb genomics platform on the PubMLST website (http://pubmlst.org/neisseria) (*12*). Four STs, all belonging to cc11, were identified: ST-11 (n = 11), ST-1287 (n = 2), ST-3298 (n = 1), and ST-12351 (n = 5). All isolates had the same PorA:FetA profile (P1.5,2:F1–1) as that identified in the MenW:cc11 collection responsible for outbreaks in South America and the United Kingdom (*8*). Furthermore, genomic sequences indicated the isolates from Western Australia were within the same United Kingdom–South America cluster as isolates from the eastern coast of Australia (*13*).

**Figure 1:**
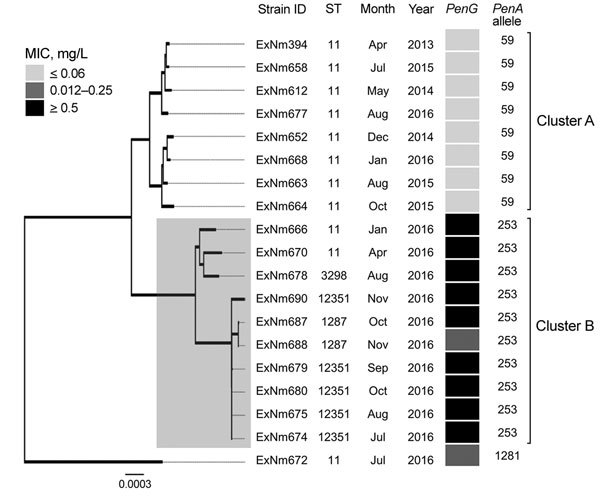
Neighbor-joining dendrogram (500 bootstrap values) for core genome sequences of clonal complex 11 *Neisseria meningitidis* strains with serogroup W capsules, Western Australia, Australia, January 2013–December 2016. The resistance phenotype for the penicillin G (*PenG*) gene for each isolate is provided using the following breakpoints: sensitive (MIC <0–0.06 mg/L), intermediate (0.12–0.25 mg/L) and resistant (>0.5 mg/L). Two clusters (A and B) were observed, which contain isolates that differ in penicillin resistance profile. Of 1,605 core-genome loci, a minimum of 244 are different between clusters A and B. The more recent cluster B appeared in early 2016 and contains penicillin-resistant isolates. Strain ExNm672 does not belong to either cluster. The dendrogram is drawn to scale, and sum of branch lengths between 2 strains indicates the proportion of nucleotide differences between those core genomes (≈1.5 Mb) within the pairwise alignment. Gray shaded box indicates isolates in cluster B. Scale bar indicates nucleotide substitutions per site. ID, identification; ST, sequence type.

Phylogenetic analysis of the meningococcal core genome (*14*) identified 2 distinct clusters within the MenW:cc11 population of Western Australia (Figure 1). One isolate (ExNm672) was an outlier and could not be clustered. ExNm672 was isolated from a traveler from Asia who had recently arrived in Western Australia, which would likely explain the different genealogy of this strain. All isolates less susceptible to or resistant to penicillin were in cluster B. Geocoding analysis showed that the 10 isolates in cluster B were obtained from 7 geographically well-separated regions in Western Australia. This observation suggests successful expansion of a new penicillin-resistant clone in 2016.

For *N. meningitidis*, polymorphisms within the gene encoding PBP2, also known as *penA*, are associated with a reduced affinity, and thus a decrease in susceptibility, to penicillin. All isolates in cluster A had the *penA_59* allele, and isolates in cluster B had the *penA_253* allele. These alleles differ by 101 nt, and the encoded peptides differ at 25 aa positions. The different amino acid residues are located in the second half of the protein, which contains the transpeptidase domain for penicillin binding. Six of the amino acid mutations encoded by *penA_253* (F504L, A510V, N512Y, I515V, H541N, and I566V) have been reported to be associated with decreased susceptibility to penicillin (*15*).

**Figure 2:**
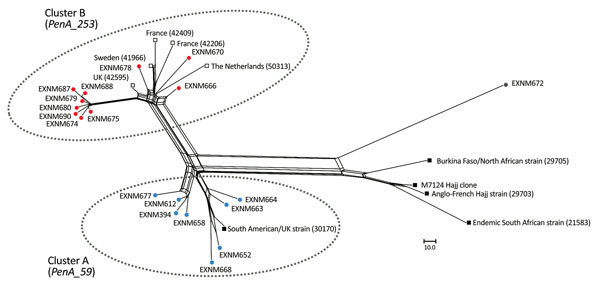
Phylogenetic reconstruction by using an unrooted neighbor-net algorithm of core genomes of clonal complex 11 *Neisseria meningitidis* strains with serogroup W capsules (MenW:cc11), Western Australia, Australia, January 2013–December 2016. Blue circles indicate isolates in cluster A from Western Australia; red circles indicate isolates in cluster B from Western Australia; gray circle indicates ExNm672, a strain isolated from a traveler; open squares indicate the 5 MenW:cc11 isolates in the PubMLST database (http://pubmlst.org/neisseria) that contains the *penA_253* allele; and black squares indicate reference MenW:cc11 strains, isolated after 2010, as described by Lucidarme et al. (*8*). M7124 is the Hajj clone isolated in Saudi Arabia in 2000. Numbers in parentheses indicate PubMLST numbers of reference isolates. Scale bar indicates nucleotide substitutions per site.

The *penA_253* allele was identified in MenB isolates of the cc32 lineage in Europe in early 2012. The PubMLST database has 5 MenW:cc11 invasive isolates harboring this allele, all of which were obtained in Europe in 2016: 2 from France, 1 from Sweden, 1 from the United Kingdom, and 1 from the Netherlands. Core-genome analysis showed clustering of these isolates from Europe with cluster B isolates from Western Australia. (Figure 2).

To assess whether *penA* was responsible for the difference in penicillin resistance between the 2 clusters, the *penA_253* allele from cluster B was transformed into all 8 penicillin-sensitive isolates in cluster A. We subsequently tested the *penA_253* isogenic mutants obtained for penicillin resistance by using the Etest. All transformants displayed intermediate resistance to penicillin (4-fold increase in MIC to 0.25 mg/L). These results indicate that the *penA_253* allele plays a major role in increased resistance to penicillin among cluster B isolates.

However, exchange of *penA* did not fully account for the level of resistance displayed by drug-resistant clinical isolates. This finding suggests that acquisition of penicillin resistance among cluster B isolates is multifactorial. Because PBP1, PBP3, and PBP4 were identical in all isolates in clusters A and B, there must be additional as yet undetermined factors that play a role in conferring resistance to penicillin in cluster B isolates. A comparison of the core and accessory genomes of isolates in the 2 clusters is required to further elucidate this issue.

## Conclusions

MenW is now the predominant serogroup causing IMD in Western Australia. Core-genome analysis identified a new cluster of penicillin-resistant MenW:cc11 clinical isolates that emerged throughout this region during early 2016. We demonstrated that the *penA_253* allele has a major role in increasing penicillin resistance among isolates in this new cluster. Because *penA_253* has been identified in MenW:cc11 isolates in Europe in 2016, jurisdictions are encouraged to monitor emergence of strains harboring this allele by PCR for culture-negative cases.
